# Iranian crack induces hepatic injury through mitogen-activated protein kinase pathway in the liver of Wistar rat 

**DOI:** 10.22038/IJBMS.2018.23543.5930

**Published:** 2018-11

**Authors:** Aliasghar Parvaresh Anbar, Tayyebeh Piran, Mehrdad Farhadi, Pouran Karimi

**Affiliations:** 1 Higher Academic Education Institute of Rab-e Rashid, Tabriz, Iran; 2 Neurosciences Research Center, Tabriz University of Medical Sciences, Tabriz, Iran

**Keywords:** Cytokines, c-JNK, Iranian Crack, Liver fibrosis, p38 MAPK, Transaminase

## Abstract

**Objective(s):**

Iranian crack (IC) is a heroin-based substance manifesting various pathologic side effects. Herein, we aimed to investigate the mechanism of IC-induced liver injuries in Wistar rats.

**Materials and Methods:**

Twenty male Wistar rats were randomly divided into two groups: control, and IC (0.9 mg/kg/day/IP, for 30 days). Mitochondrial reactive oxygen species (ROS) production was measured by DCF fluorescence staining. The expression of tumor necrosis factor-alpha (TNF-α), interleukin 1β (IL-1β), and phosphorylation of p38 mitogen-activated protein kinase (p38 MAPK) and c-Jun N-terminal kinase (c-JNK) were assessed by immunoblotting assay. The intensity of collagen fiber in the liver was also determined by Trichrome-Masson staining. Furthermore, serum levels of alanine aminotransferase (ALT), aspartate aminotransferase (AST), and alkaline phosphatase (ALP) activities were measured using colorimetric methods.

**Results:**

Our results showed that ROS production, p38 MAPK, c-JNK phosphorylation levels, and expression of TNF-α and IL-1β were significantly elevated in the liver tissue of IC group as compared to the control group. Moreover, collagen fiber and ALT activity were increased in the liver tissue of IC group compared to the control group. However, there was no statistically significant difference in the levels of ALP between two groups. In addition, there was a positive correlation between the intensity of collagen fiber and the ALT activity, and the levels of TNF-α and IL-1β and liver enzymes activities including ALP, ALT, and AST.

**Conclusion:**

Our findings revealed that IC-induced liver cells injury is partially mediated by MAPK stress kinases. Therefore, regular liver examination in substance abuse is strongly recommended.

## Introduction

Opiates addiction is a major public health concern worldwide particularly in developing countries (1, 2). Recently, a newfound heroin-based narcotic has been extensively used in Iran named crack (3, 4). Based on evidence, Iranian crack (IC) is completely different from the common crack cocaine (3, 5). The prevalence of crack abuse in Iran is unknown; however, given its ease of use, simple and rapid preparation and inodorousness, this drug may be very common in Iran (3). The major ingredients of IC are morphine, caffeine, codeine, thebaine, acetaminophen, and a significant amount of acetyl codeine (3, 4). Although physiological and psychological effects of this narcotic have been partially studied, its somatic damage is far less investigated (6, 7). 

Regarding the function of liver in drug metabolisms, the liver is the most pre-exposed organ to IC-induced injury (2). The most common features of liver damage (LD) are the accumulation of extracellular matrix proteins such as collagen, so-called liver fibrosis, inflammation and increased aminotransferases activity (2, 8-12). Liver fibrosis is a tissue-repairing response to chronic liver injury, which results from the hepatic stellate cell stimulation (13-15). Upon liver injury, the stellate cells proliferate and produce a large amount of collagen-rich matrix (10, 12, 16, 17). Following LD, parenchymal tissue regeneration pathways are triggered by mitogen-activated protein kinases (MAPKs) including the p38 MAP kinases and the c-jun N-terminal kinases (JNKs). Among them, JNK is activated during the first hours following LD (18). Nevertheless, p38 MAPK phosphorylation is elevated within 1-3 days after partial hepatectomy in rats in order to control the activity of gap and tight junctions during the liver regeneration (19). Hepatic cells damage can also be evaluated by groups of blood enzymes activity tests (20). It is generally accepted that the degree of the liver cell membrane and mitochondrial damage can be monitored by evaluation of aminotransferase activity. Alkaline phosphatase (ALP) activity is an indicator for cholestatic damages of the liver (21). 

Moreover, pro-inflammatory cytokines such as tumor necrosis factor-alpha (TNF-α) and interleukin 1β (IL-1β) are produced by several cell types including initiate immune response- related cells such as lymphocytic natural killer (NK) cells, Kupffer cells (KCs), hepatic parenchymal cells, hepatic stellate cells (HSCs), and dendritic cells (DCs). Cytokines have an immunoregulatory role in all over the body (22, 23). Pro-inflammatory cytokines have also been extensively studied as a hepatic injury indicator (1, 22, 24). 

Despite the common use of IC in Iran, there is no study focusing on its pathologic effects on liver tissue. The current study aimed at identifying the effect of chronic administration of IC on hepatic cells injury in Wistar rats.

## Materials and Methods

***Drug and antibodies***

IC was obtained from Dr. Amoghli Tabrizi. The TNF-α Antibody (N-19): SC-1350, Anti**- IL-**1β (E7-2-HILΒ): SC-32294, Anti-**p-p38** Antibody (Thr 180/Tyr 182): sc-17852-R, p38α Antibody (C-20): sc-535, p-JNK (G-7): sc-6254, **JNK **Antibody (FL): sc-571, Anti- β-actin (sc-130656) and HRP-conjugated goat Anti-rabbit secondary polyclonal antibody (SC-2030) were purchased from Santa Cruz (Santa Cruz Biotechnology, Inc., CA, USA).

***Ethics statement***

This study was approved by the Ethics Committee of Higher Academic Education Institute of Rab-e Rashid, Tabriz, Iran (Approval number: 3455J666) based on recommendations and the policies of the International National Institutes of Health (NIH) guidelines for use and handling of laboratory animals.

***Animals***

The experiment was carried out on 20 male Wistar rats, 2 months of age and weighing 250 ± 25 g, obtained from the animal facility of Tabriz University of Medical Sciences, Tabriz, Iran. Animals were kept in a standard condition (23 ± 2 °C temperature, 50-55% humidity with 12 hr light/dark cycle) and fed standard rat chow diet and water *ad libitum* (22). 

***Drug administration***

After 2 weeks of adaptation, rats were randomly allocated into two experimental groups (n=10 per group) and intraperitoneally received treatments; i) saline (0.9% NaCl, 200 µl) in the control group (NS), and ii) IC (0.9 mg/kg/day) dissolved in saline for 30 days in the IC group.

***Sampling***

At the end of the experiments, animals were anesthetized with IP injection of 90 mg/kg ketamine and 9 mg/kg xylazine mixture, and whole blood samples were subsequently collected in EDTA tubes. The blood samples were placed at room temperature for 1 hr and centrifuged at 3500 rpm for 10 min to obtain the serum. Moreover, the liver tissue was collected and stored at -80°C for further investigation. 

***Measurements of enzymes activity***

Serum levels of liver functional transaminases including alanine aminotransferase (ALT), aspartate aminotransferase (AST), and ALP were determined with a clinical auto-analyzer (LX20-Pro, Beckman-Coulter) using specific standard kits (Ziest Chimi) (2, 21).

***ROS production assay***

To determine liver reactive oxygen species (ROS) production, dichlorodihydrofluorescein diacetate (DCFDA) dye was used. For this purpose, the liver homogenate supernatant was incubated with 2 µM DCFDA for 20 min. ROS production was monitored by determining the fluorescence intensity using a fluorescent plate reader in which excitation and emission wavelengths were set at 504 and 529 nm, respectively. The ROS levels were presented as fluorescence intensity/mg protein (25).

***Histopathological examination***

The liver tissues were fixed in normal 10% neutral buffered formalin immediately, then dehydrated in a graded alcohol, and embedded in paraffin wax. Thereafter, 4 μm thickness sections were stained with hematoxylin and eosin (H&E) and Masson’s Trichrome (MT) according to the standard protocols to examine histopathology changes in the liver and collagen deposition, respectively. Images were acquired by light microscopy (Nikon Eclipse TE2000-U, Nikon, Japan), and the degree of liver fibrosis was quantified using Image-Pro Plus 6.0 software. The criteria used for scoring fibrosis severity were listed as follows according to the method as previously described (Table 1) (21, 26-28). 

***Immunobloting assay***

Western blotting was used for determining the expression of TNF-α and IL-1β proteins as well as phosphorylation of p38 MAPK and JNK in the liver tissue samples. Briefly, hepatic samples were lysed for 30 min using radioimmunoprecipitation assay (RIPA) buffer (Sigma-Aldrich) supplemented with a protease inhibitor cocktail (Roche, Germany), 1 µM phenylmethylsulfonyl fluoride (PMSF) and 0.15% β-mercaptoethanol (Sigma-Aldrich, Germany). Samples were centrifuged at 12,000 rpm for 10 min at 4 °C, and total protein concentrations were measured using Bradford reagent (Bio-Rad, USA). About 50 μg of total protein was resolved on 10% SDS-PAGE Bolt® Bis-Tris plus MES gels (Sigma-Aldrich, Germany). Then, proteins were transferred to a PVDF membrane (Sigma-Aldrich), blocked with non-fat dry milk (Sigma-Aldrich) for 30 min at room temperature, and immunolabeled with a primary antibody (anti-TNF-α, anti-IL-1β, anti-p38 MAPK and anti-JNK with β-actin as endogenous control) diluted in Tris-buffered saline (TBS) pH 7.5 at 4 °C overnight. The protein bands were visualized by high sensitivity ECL chemiluminescence kit (Bio-Rad, USA). Band densitometric analysis was performed using Image J software (National Institutes of Health, Bethesda, Maryland, USA), and band intensities were corrected for equal β-actin loading. Intensities were provided relative to the intensities of controls (29).

***Statistical analysis***

Statistical analyses were performed using SPSS 19.0 statistical software. All data were presented as mean ± SEM. The significance of difference was evaluated with Student’s t-test. The correlation coefficient of Pearson was performed between various parameters. The* P*-value less than 0.05 was considered statistically significant.

## Results

***Effects of IC on the hepatic mitochondrial ROS production***

**Figure 1:**
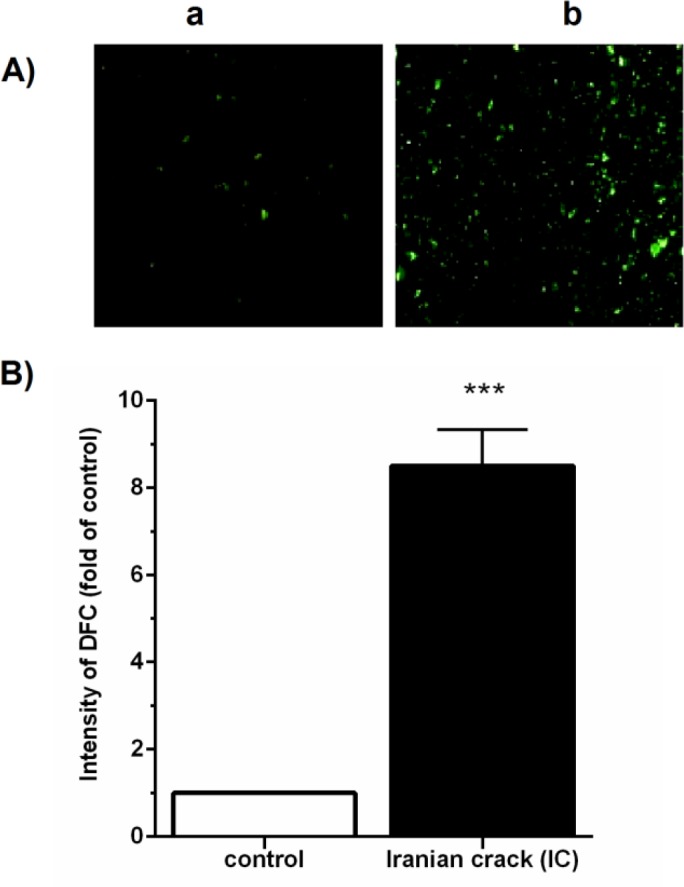
Effects of Iranian crack (IC) on the hepatic mitochondrial reactive oxygen species (ROS) production. (A) Fluorescent microscopic images of DCF-stained mitochondria in the liver tissue. (Aa) normal saline control group; (Ab) IC-received (0.9 mg/kg/day) group. (B) Quantified DCF fluorescence by Fluorimeter (Biotek), which was normalized against mg protein in mitochondrial extract and presented as fold of control per group. Values are represented as mean ± SEM (n=5); ***P<0.001 vs. control group

As shown in Figure 1, the intensity of DCF fluorescent in the IC-treated group was significantly (*P*<0.001) greater than those in the saline-treated control group.

***Effects of IC on the phosphorylation of p38*** ***MAPK and c-JNK***

**Figure 2:**
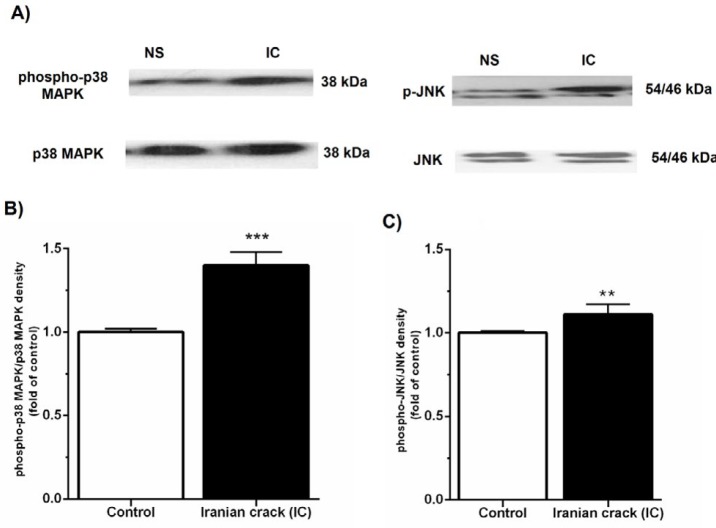
Effects of Iranian crack (IC) on the phosphorylation of p38 MAPK and JNK in the liver tissue. (A) Immunoblotting images of phosphor-p38 MAPK, total p38 MAPK, phosphor-JNK and total JNK proteins in normal saline (NS)-received rat and IC-treated rats. (B) Densitometry analysis of phosphor-p38 MAPK normalized against total p38 MAPK and presented as fold change of control. (C) Densitometry analysis of phosphor-JNK normalized against total JNK and presented as fold change of control. Values are represented as mean±SEM; **P<0.01, ***P<0.001 vs. control group

The results of Western blotting indicated that phosphor-p38 MAPK (*P*<0.001) and phosphor-JNK (*P*<0.01) proteins were significantly increased in the IC-treated rats as compared to the control animals (Figure 2).

***Effects of IC on the hepatic pro-inflammatory cytokines levels***

**Figure 3:**
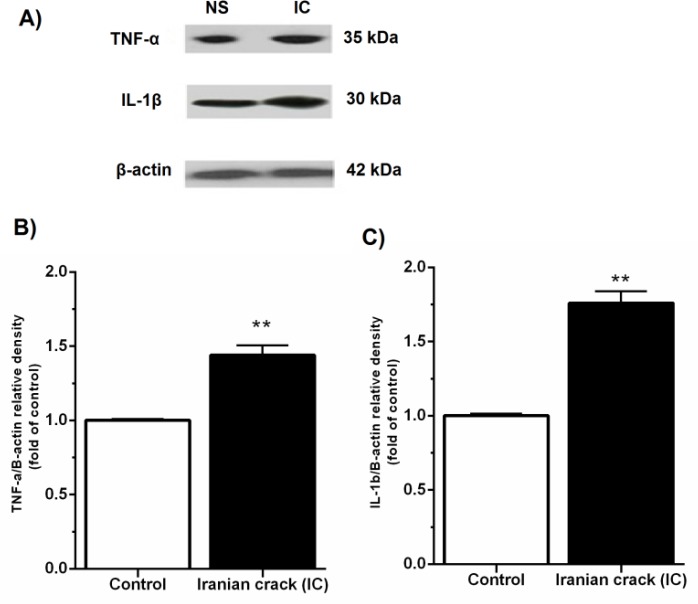
Effects of Iranian crack (IC) on the expression of tumor necrosis factor-alpha (TNF-α) and interleukin 1β (IL-1β) in the liver tissue. (A) Immunoblotting images of TNF-α, IL-1β, β-actin proteins (as the internal control) in the normal saline (NS)-received rat and IC-treated rats. (B) Densitometry analysis of TNF-α normalized against β-actin and presented as fold change of control (C) Densitometry analysis of IL-1β normalized against β-actin and presented as fold change of control. Values are represented as mean ± SEM; **P<0.01 vs. control group

Our result showed that chronic administration of IC is associated with up-regulation of TNF-α and IL-1β proteins in the liver (Figure 3).

***Effects of IC on serum AST, ALT, and ALP enzyme activities in rats***

Biochemical parameters such as AST, ALT, and ALP were assessed as reflections of the liver destruction (30) and determined by the colorimetric methods.

The results presented in Table 2 indicated that IC caused a significant increase (*P*<0.01) in serums ALT and AST levels in rats. However, serum levels of ALP did not show any significant changes when compared to the control group (Table 2).

***Effects of IC on histopathological features in the liver tissue***

**Figure 4:**
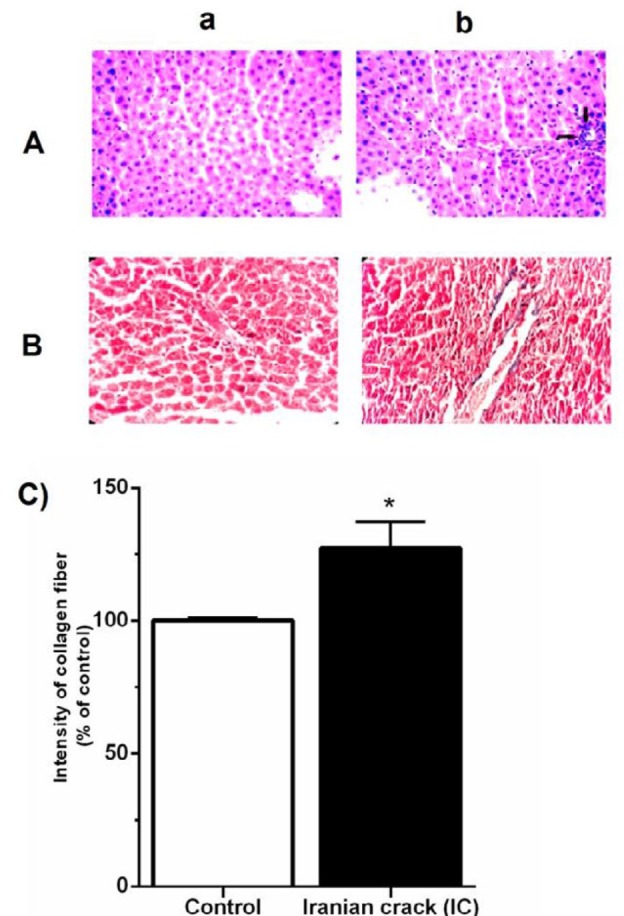
Effect of Iranian crack (IC) on the liver tissue inflammation and fibrosis. A) 400× hematoxylin and eosin (H&E)- stained sections and B) Trichrome Masson (MT)-stained sections. (Aa) Photomicrographs of liver sections of normal control group showed well-structured and arrangement of the normal liver architecture and few inflammatory cell infiltration; (Ab) Photomicrographs of liver sections of IC-administered group showed inflammation and mononuclear cell infiltration in the liver tissue (black arrow); (Ba) the rate of collagen as a marker of fibrosis in normal saline control group (blue color) (Bb) representative of the collagen and other fibrotic agents accumulation in the liver tissue of IC-treated rats (blue color). (C) The score of liver fibrosis in different groups. Values are presented as mean ± SEM (n=5); *P<0.05 vs. control group

The results of light microscopic studies on liver sections in the control group stained with H&E and MT indicated that the hepatocytes were arranged in strands with one or two spherical nuclei, and sinusoids were occupied by blood cells. In addition, the cytoplasm of hepatic cells was slightly eosinophilic and the central vein had a circular outline (Figure 4 Aa, Ba). Nevertheless, IC group showed pronounced morphological alterations including disruption of the tissue architecture, extension of portal tract with infiltration of inflammatory cells, increased necrotic cells among the hepatocytes of boundary wall and lobule, and the major structural component (i.e., collagen) (Figure 4 Ab, Bb). Moreover, IC-treated group showed significantly (*P*<0.05) higher portion of collagen than the control group (Figure 4C).

***Correlation among the hepatic inflammatory cytokines, serum aminotransferase, and ALP enzymes activity and the intensity of collagen fiber in the IC group***

The results of correlation between inflammatory cytokines, serum aminotransferase and ALP enzymes activity, and intensity of collagen fiber in the IC group are illustrated in Table 3. The results showed that elevated levels of inflammatory cytokines, including TNF-α and IL-1β were positively correlated with increased serum ALP, ALT, and AST activities. In addition, there was a positive correlation between the intensity of collagen fiber and the levels of ALT in the IC group.

**Table 1:** The criteria for scoring fibrosis severity

Score	The fibrosis exist in central vein and Sinus (L)	The scope of fibrosis in portal area (P)	Fiber spacing (s)	
			Width (W)^a^	Amount (N)
0	none	expanded,	none	-
1	limited, a few	no bridging	<6/cm	Thin
2	diffused, most	>6/cm	>6/cm	loose, broad
3	-	Cirrhosis	cirrhosis	condensed, broad
4	–	-	≥2/3	Biopsy

Scoring: *L+ P+2(N×W)*;

a Only a thin fibrous septa in the sample, *W* scored as 0.5.

**Table 2:** Effects of Iranian crack (IC) on serum alanine transaminase (ALT), aspartate aminotransferase (AST) and alkaline phosphatase (ALP) enzyme activities in rats

Groups	ALT (U/L)	AST (U/L)	ALP (U/L)
Control	22.430±5.259	66.620±17.754	85.890±27.914
IC	82.433±21.040**	316.74±149.116**	119.46±46.185

Values are expressed as mean ± SEM.

** *P*<0.01 vs. control group.

**Table 3:** Correlation between inflammatory cytokines, serum enzymes and intensity of collagen fiber in the IC group

**Variables**	**Analysis**	**ALT** **(U/L)**	**AST** **(U/L)**	**ALP** **(U/L)**
**TNF-α**	Spearman. c. c	0.863**	0.733**	0.740**
	*P* Value	0.000	0.004	0.004
**IL-1β**	Spearman. c. c	0.837**	0.750**	0.594*
	*P* Value	.000	0.003	0.032
**CF%**	Spearman. c. c		0.755*	
	*P* Value	ns	0.030	ns

ALT: Alanine aminotransferase; AST: Aspartate aminotransferase; ALP: Alkaline phosphatase; TNF-α: Tumor necrosis factor-α; IL-1β: Interleukin 1 beta; CF% percentage of collagen fiber; c.c correlation coefficient.

* *P*< 0.05,

** *P*< 0.01 vs. control group

## Discussion

To our knowledge, this is the first experimental study to investigate the effect of chronic administration of IC on the liver injury in rats. The main findings of the current study were as follows: IC led to i) hepatic mitochondrial ROS overproduction, as confirmed by elevated DCF fluorescence intensity in hepatic isolated mitochondria ii) hyperphosphorylation of P38 and JNK MAPK proteins, iii) increased hepatic expression of TNF-α and IL-1β and accumulation of collagen fibers, and iv) increased serum levels of ALP, ALT, and AST. 

IC is a heroin-based opiate with major components of morphine, caffeine, codeine, and thebaine, which is indirectly metabolized to morphine (3). Despite numerous studies on the addictive properties of IC (30-32), there are a few reports about the cytopathologic effects of IC on body organs in general and the liver in particular in abusers. The liver is the major site of metabolism for the most drugs, especially opiates (6, 7). 

The central role of the liver in drug biotransformation prompts it to toxic damage so-called hepatotoxicity, which is associated with the formation of free radicals and oxidative stress, inflammation, fibrosis and liver cell destruction (2). Hepatocytes have enzymatic and non-enzymatic antioxidant defenses to remove or neutralize ROS (34). Overproduction of ROS may overwhelm hepatocellular antioxidant defenses and lead to the DNA, protein and unsaturated fatty acids damage and cell death (7, 35-40). Moreover, it has been reported that heroin, morphine, and opiates reduce antioxidant defense (34, 40). In this study, we found that chronic administration of IC increased ROS production in the liver. Similarly, Samarghandian *et al.* reported that morphine increases endogenous lipid malondialdehyde levels and decreases enzymatic antioxidant activities (2). 

Furthermore, previous studies showed that ROS, and in particular H_2_O_2,_ are required for inflammatory cell recruitment (41) and activation of the stress-activated MAP kinases p38 and the Jun-N terminal kinase (JNK) (42, 43). Activation of MAPKs pathway initiates the transcription of pro-inflammatory cytokines, which are a group of important regulatory mediators involved in the development of liver injury (44). It has also been proven that during infection, tissue damage and different stresses, the nuclear transcription factor NF-kappa B is activated and translocated to the nucleus and activates MAPKs such as p38 and JNK (8, 45), which in turn increases transcription of numerous inflammatory-related genes and increases synthesis of a range of proteins involved in the inflammatory response (45-47). 

Some studies have revealed that opiates, specifically heroin and morphine, disrupt immunocompetence (1, 22), and increase the production of certain cytokines within a few minutes after morphine administration (1). Moreover, the extreme TNF-α activity can result in tissue toxicity and damage (48). The hepatic macrophage-derived pro-inflammatory cytokines such as TNF-α and IL1β can affect activated-hepatic stellate cells and, through NFκB activation, promote the survival of hepatic stellate cell-derived myofibroblasts and the development of liver fibrogenesis (49, 50). In the current experiment, IC induced hyperphosphorylation of p38 MAPK and JNK in the liver accompanying with up-regulation of TNF-α and IL-1β proteins.

The AST, ALT, and ALP are the most important and effective indexes for evaluating liver cell damage and cholestasis, and abnormal liver enzyme levels may indicate liver damage (21, 28, 30). Chronic hepatitis is a common outcome found in heroin addicts (51). A previous study reported that long-term treatment with morphine increases the serum levels of ALT, AST, and lactate dehydrogenase (LDH) enzymes in the rat (2). Our results also indicated that IC caused a significant increase in serums ALT and AST levels in rats, although serum levels of ALP did not show significant changes compared to the control group. Moreover, we found that elevated levels of inflammatory cytokines (TNF-α and IL-1β) were positively correlated with serum enzymes (ALP, ALT, and AST) in IC group, which is further demonstrating the applicability of these enzymes in monitoring the liver inflammation. 

In this study, histopathological analysis of the liver section in the IC group also showed pronounced morphological alterations, as evidenced by disruption of the tissue architecture, extension of portal tract with infiltration of inflammatory cells, increased necrotic cells among the hepatocytes of boundary wall and lobule, and collagen deposition. In addition, there was a positive correlation between the intensity of collagen fiber and serum levels of ALT.

Hepatic fibrosis is an outcome of liver injury, which results in activation of collagen-producing cells and extreme deposition of extracellular matrix (ECM) proteins as a part of the tissue repair response to chronic liver injury (13-15). Upon liver injury, the stellate cells proliferate and produce a large amount of collagen-rich matrix. Previous studies reported that the chronic use of morphine induces hepatic portal tract fibrosis, bile ductal dilatation, and proliferation (16, 52). Another research in a group of intravenous heroin addicts demonstrated hyperplasia and hypertrophy of the smooth endoplasmic reticulum (SER), a vesicular degeneration of hepatocyte developed as a result of the increased synthesis of enzymes of SER, and the presence of continuous basal membrane followed by conversion of the sinusoids into capillaries (53). In this study, histopathology examinations demonstrated considerable accumulation of collagen fiber in the liver tissue of IC-received rats.

## Conclusion

Our findings pointed out the risk of increased hepatic cell destruction, liver fibrosis, and inflammation following chronic use of IC. 
